# Characteristics of safety information obtained from postmarketing observational studies for re-examination in Japan

**DOI:** 10.1186/s40064-016-2365-4

**Published:** 2016-06-27

**Authors:** Tatsuya Watanabe, Mamoru Narukawa

**Affiliations:** Department of Clinical Medicine (Pharmaceutical Medicine), Graduate School of Pharmaceutical Sciences, Kitasato University, 5-9-1 Shirokane, Minato-ku, Tokyo, 108-8641 Japan; Department of Research, Kitasato University Kitasato Institute Hospital, Tokyo, Japan

**Keywords:** Postmarketing surveillance, Pharmacovigilance, Package insert, Re-examination, Safety

## Abstract

**Background:**

In Japan, postmarketing surveillance (PMS) studies are required for newly approved drug products to further collect safety information in clinical settings. “PMS study” is a general term encompassing both postmarketing observational (PMO) studies and postmarketing intervention studies for re-examination. Each PMS study is conducted under contracts between the pharmaceutical company and medical institutions in accordance with Good Postmarketing Study Practice. It has been reported that the safety information collected postmarketing is limited because of underreporting. The objective of this investigation was to identify differences among profiles of the drug product safety information collected through intervention studies and observational studies before and after approval. Our study addressed whether the issue of underreporting, generally considered as associated with observational studies, occurs in PMO studies for re-examination. In addition, we considered potential causes of such underreporting.

**Results:**

The overall adverse reaction rate was lower in PMO studies than in intervention studies before approval in almost all cases. The adverse reaction rate in intervention studies exhibited similar profiles regardless of whether they were conducted prior to or following approval. In addition, we found that one reason for a lower adverse reaction rate in PMO studies was that the number of reports of adverse reactions that had occurred frequently prior to approval decreased postmarketing.

**Conclusions:**

Underreporting was observed even in PMO studies for re-examination under the Japanese regulation. Although it was suggested that expected and common adverse reactions were more likely to be subject to underreporting, further investigation is warranted to explore the reasons for the under-reporting in PMO studies.

## Background

Information on drug product safety is collected beginning before marketing approval and continuing after approval. Before approval, highly accurate information can be obtained through intervention studies (phase 1, 2, and 3 clinical trials) conducted in specific populations. After approval, safety information is primarily collected based on spontaneous reports and/or observational studies performed in daily medical examinations (Banerjee et al. 2013). Generally, in intervention studies, randomized, double-blind, comparative studies intended to verify hypotheses are conducted to obtain results with a high level of evidence. In contrast, in observational studies—sometimes beneficial for forming hypotheses because of potentially large sample numbers—control groups are virtually never included and the level of evidence of the results, therefore, is not very high.

Clinical studies prior to approval are usually conducted under a limited range of conditions, which has been referred to as the “5 toos” (too few, too simple, too narrow, too median-aged and too brief) (Rogers 1987) making it difficult to obtain all necessary safety information solely with such studies. In recent years, there has been more internationalization of clinical drug development programs. This has resulted in approval in Japan of new drug products for which only limited safety information on Japanese patients had been collected and evaluated (Iwasaki et al. 2012). Therefore, “collection of safety information at the postmarketing stage especially through observational studies” is becoming increasingly important. However, it has been reported that safety information collected postmarketing is limited because of underreporting (Hazell and Saad 2006; Macdougall et al. 2008). It is generally understood that expected and non-serious adverse reactions are more likely to be underreported. Bäckström et al. (2004) showed that at least 80 % of adverse reactions occurring postmarketing are not reported. Lopez-Gonzalez et al. (2009) attributed underreporting to such causes as “ignorance/preconceptions” (a belief that only serious adverse reactions need to be reported) and a “sense of security” (a feeling that only safe drug products are allowed onto the market).

Regarding regulation of postmarketing, in the European Union (EU) postmarketing safety monitoring systems have been strengthened, including by pharmacovigilance legislation implemented in 2012 (European Union 2011). In Japan as well, a guideline for a drug risk management plan (RMP) was issued in 2012 and, since April 2013, companies applying for marketing approval have been required to submit a RMP that contains postmarketing pharmacovigilance and risk minimization plans. These plans must account for risks, such as important identified/potential risks and missing information of the drug product (Risk Management Plan 2012). Japan is currently in the process of developing a new pharmacovigilance system.

In Japan, postmarketing surveillance (PMS) studies are required for newly approved drug products to ensure further collection of safety information in clinical settings. “PMS study” is a general term that encompasses both postmarketing observational (PMO) studies for re-examination, treatment outcome studies and postmarketing intervention (PMI) studies for re-examination, also known as phase 4 clinical studies. Each PMS study is conducted under contracts between the pharmaceutical company and medical institutions in accordance with Good Post-marketing Study Practice (GPSP) Ministerial Ordinance (MHLW Ministerial Ordinance No. 171, issued December 20, 2004).

For most new drug products or existing drug products for which additional indications have been approved, PMO studies for re-examination are routinely conducted to collect safety information after approval. “Re-examination” is a regulatory system specifying safety and efficacy testing of marketed new drugs within a certain period of time (normally 8 years) after approval to re-confirm drug effectiveness in clinical settings. This effectiveness is based primarily on the results of PMO studies for re-examination and spontaneous reports of adverse reactions. Because of this system, many PMO studies for re-examination are conducted postmarketing and are sponsored by pharmaceutical companies. However, most of these PMO studies for re-examination are conducted in a target sample size of 3000 patients without a control group. This sample size is regarded as sufficient to detect, with a 95 % probability, relatively rare adverse reactions occurring in about 1 out of 1000 patients (an incidence of 0.1 %) (Iwasaki et al. 2012). Neither the sample size nor the study purpose takes into account the characteristics of the safety information that was collected prior to drug product approval. For this reason, the safety information obtained from PMO studies for re-examination are seldom used for postmarketing safety actions such as revision of package inserts (Kanmuri and Narukawa 2014).

Conversely, in PMI studies for re-examination, the objectives of which may include collecting additional information not collected in the clinical studies conducted prior to approval, control groups are established and randomization is often performed. However, such PMI studies for re-examination are conducted only occasionally and for a limited number of new drugs. In fact, according to the ClinicalTrials.gov registry, there are few industry-funded PMI studies for re-examination being conducted in Japan.

Information collected in PMO studies for re-examination is structured, unlike that in spontaneous reports, and study plans are submitted in advance to the Ministry of Health, Labour and Welfare. In addition, PMO studies are conducted by medical professionals under contracts signed between the pharmaceutical companies and medical institutions. Therefore, PMO studies for re-examination are expected to collect safety data with higher quality and credibility compared with data collected under other observational studies. Nonetheless, in a questionnaire survey regarding PMO studies for re-examination administered to medical representatives of pharmaceutical companies, the representatives reported that non-serious and expected adverse reactions in particular are often underreported (Watanabe and Narukawa 2015). It was also reported that in PMO studies for re-examination, data collection is performed primarily by the investigators themselves with little support from other departments (Watanabe and Narukawa 2014).

With this as background, we focused on safety data collected in PMO studies in Japan, which are routinely conducted under the framework of the pharmaceutical regulation known as re-examination. The objective of our investigation was to identify differences in profiles of the drug product safety information collected through intervention studies and observational studies, and before and after approval. Our study addressed whether the issue of underreporting, generally considered as associated with observational studies, occurs in PMO studies for re-examination. In addition, we considered potential causes of such underreporting.

## Methods

### Extraction of analysis sets

When a re-examination is completed for a product, its package insert is revised based on the results of PMS studies. This revision is considered a “milestone revision,” making it possible to comprehensively identify the postmarketing safety information that was collected, primarily by the pharmaceutical company.

We searched the package inserts of drug products for which re-examinations were completed between January 2009 and December 2014. Among them, we identified package inserts for 189 drug products that included information categorized as “adverse reaction rate in clinical studies for new drug application (NDA)” and “adverse reaction rate in PMS studies” and used these inserts for our investigation. We also extracted information listed under “adverse reaction rate in PMI studies for re-examination,” either from the package insert or the re-examination report of the product. We used the package insert search tool of the Pharmaceuticals and Medical Devices Agency to obtain package inserts, re-examination reports and drug product interview forms for the drug products included in our analysis.

### Information extraction and tabulation

#### Comparison of overall adverse reaction rates before versus after approval

From the package inserts of the aforementioned 189 drug products, we extracted the overall adverse reaction rate in clinical studies for NDA (ARR-NDA) and the overall adverse reaction rate in PMO studies for re-examination (ARR-PMO) and prepared a scatter plot. Furthermore, for drug products for which PMI studies for re-examination were conducted following approval, we extracted the overall adverse reaction rates in PMI studies for re-examination (ARR-PMI) and prepared a scatter plot against those for the ARR-NDA.

The number of ARR-NDA and ARR-PMO were calculated in the following way.

- ARR-NDA (%) = total number of all adverse reactions/total number of subjects in clinical studies for NDA
- ARR-PMO (%) = total number of all adverse reactions/total number of subjects in PMO studies for re-examination.

#### Comparison of incidence rates of the most common adverse reactions in clinical studies for NDA versus in postmarketing observational studies for re-examination

For each of the 189 drug products, we specified the most common adverse reaction in clinical studies for NDA based on their package inserts and then calculated the difference between this incidence rate and that obtained in PMO studies for re-examination. We calculated the difference between overall adverse reactions data from ARR-NDA and ARR-PMO and prepared a scatter plot. Each adverse reaction for the investigation was classified by the system organ class of the Medical Dictionary for Regulatory Activities (MedDRA/J Ver.19.0J), and also categorized as serious or non-serious based on the presence or absence in the section of “serious adverse reactions” of the package insert.

## Results

We identified 189 drug products for which the information of adverse reaction rates both in clinical studies for NDA and in PMS studies were available. Among these, both the ARR-NDA and ARR-PMO were available for 176 products and both the ARR-NDA and ARR-PMI were available for 45 products. For 162 of the drug products, it was possible to compare incidence rate of the most common adverse reaction obtained in clinical studies for NDA to that obtained in PMO studies for re-examination. Characteristics of these products are shown in Table 1.

**Table 1:** Characteristics of the products investigated (176 drug products for Fig. 1, 45 drug products for Fig. 2 and 162 drug products for Fig. 3)

	(A) 176 drug products	(B) 45 drug products	(C) 162 drug products
Therapeutic group			
A—alimentary tract and metabolism	18	8	17
B—blood and blood-forming organs	9	3	8
C—cardiovascular system	20	3	20
D—dermatologicals	6	3	5
G—genitourinary system and sex hormones	13	1	13
H—systemic hormonal preparations, excluding sex hormones and insulins	7	1	6
J—anti-infectives for systemic use	25	5	23
L—anti-neoplastic and immunomodulating agents	10	3	10
M—musculoskeletal system	6	3	6
N—nervous system	24	8	23
P—anti-parasitic products, insecticides, and repellents	2	0	1
R—respiratory system	15	4	10
S—sensory organs	6	1	6
V—various	15	2	14
Re-examination dates			
January 2009 to December 2009	45	6	42
January 2010 to December 2010	44	13	42
January 2011 to December 2011	30	9	27
January 2012 to December 2012	21	5	20
January 2013 to December 2013	19	8	16
January 2014 to December 2014	17	4	15

(A) Drug products for which both the adverse reaction rate in clinical studies for NDA and in postmarketing observational (PMO) studies were available (Fig. 1)

(B) Drug products for which both the adverse reaction rate in clinical studies for NDA and in postmarketing intervention (PMI) studies were available (Fig. 2)

(C) Drug products for which the incidence rate of the most common adverse reaction in clinical studies for NDA and in PMO studies for re-examination were available (Fig. 3)

### Comparison of overall adverse reaction rates before versus after approval

First, we compared the ARR-NDA and ARR-PMO. We defined a drug product whose package inserts contained both the ARR-NDA and ARR-PMO for an individual indication as 1 set and obtained 206 sets of such information from 176 products (Table 1). We prepared a scatter plot with the ARR-NDA along the vertical axis and the ARR-PMO along the horizontal axis (Fig. 1). This plot showed that the overall adverse reaction rates in clinical studies for NDA were higher than those in PMO studies for re-examination in 88.3 % of the information sets (182 of 206 sets).

**Fig. 1:**
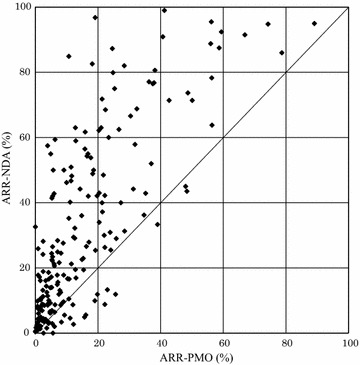
Scatter plot of the adverse reaction rate in clinical studies for NDA (ARR-NDA) and the adverse reaction rate in PMO studies for re-examination (ARR-PMO) (176 drug products, 206 sets). *Vertical axis* *ARR-NDA* adverse reaction rate in clinical studies for NDA. *Horizontal axis* *ARR-PMO* adverse reaction rate in postmarketing observational studies for re-examination

**Fig. 2:**
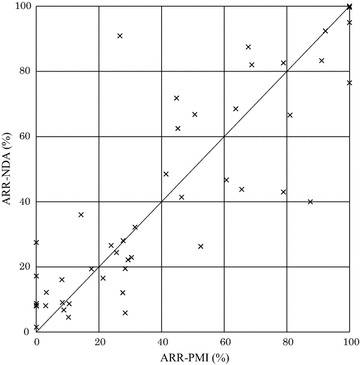
Scatter plot of the adverse reaction rate in clinical studies for NDA (ARR-NDA) and the adverse reaction rate in PMI studies (ARR-PMI) (45 drug products, 48 sets). *Vertical axis* *ARR-NDA* adverse reaction rate in clinical studies for NDA. *Horizontal axis* *ARR-PMI* adverse reaction rate in postmarketing intervention studies

We next compared the ARR-NDA and ARR-PMI. We extracted the adverse reaction rates in PMI studies for re-examination from the package insert or the re-examination reports of 189 drug products, obtaining 48 sets of ARR-NDA and ARR-PMI data for 45 products (Table 1). We prepared a scatter plot with the ARR-NDA along the vertical axis and the ARR-PMI along the horizontal axis (Fig. 2). This plot showed that the overall adverse reaction rates in clinical studies for NDA were higher than those in PMI studies for re-examination in 56.3 % of the information sets (27 of 48 sets).

### Difference of incidence rates of the most common adverse reaction in clinical studies for NDA versus in postmarketing observational studies for re-examination

**Fig. 3:**
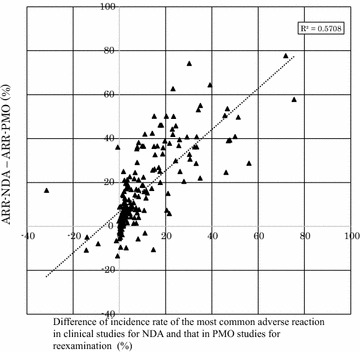
Relationship between: the *difference of incidence rate of the most common adverse reaction* in clinical studies for NDA and that in PMO studies; and the *difference of the overall adverse reaction rate* in clinical studies for NDA and that in PMO studies (162 drug products, 192 sets). *Vertical axis* difference of the *overall adverse reaction rate* in clinical studies for NDA and that in PMO studies for re-examination (ARR-NDA–ARR-PMO). *Horizontal axis* difference of *incidence rate of the most common adverse reaction* in clinical studies for NDA and that in PMO studies for re-examination

We prepared a scatter plot for the 192 sets of data obtained for 162 drug products as follows: differences between the incidence rates of the most common adverse reaction in clinical studies for NDA and those in PMO studies for re-examination on the horizontal axis, differences between the overall adverse reactions in ARR-NDA and those in ARR-PMO on the vertical axis (Fig. 3). This plot showed that the two variables were correlated (Spearman’s r = 0.800, p < 0.0001). Each adverse reaction was classified by the MedDRA system organ class (Table 2). Serious adverse reactions were observed more often in “metabolism and nutrition disorders”, “vascular disorders” and “cardiac disorders”.

**Table 2:** Characteristics of the most common adverse reaction in clinical studies for NDA (162 drug products, 192 sets for Fig. 3)

System organ class	Serious adverse reaction 20 sets	Non-serious adverse reaction 172 sets
Blood and lymphatic system disorders	1	1
Cardiac disorders	3	0
Eye disorders	0	8
Gastrointestinal disorders	0	42
General disorders and administration site conditions	1	26
Hepatobiliary disorders	2	1
Immune system disorders	1	0
Investigations	0	24
Metabolism and nutrition disorders	6	3
Musculoskeletal and connective tissue disorders	0	2
Nervous system disorders	1	30
Psychiatric disorders	0	1
Renal and urinary disorders	0	1
Reproductive system and breast disorders	0	5
Respiratory, thoracic and mediastinal disorders	0	2
Skin and subcutaneous tissue disorders	0	17
Vascular disorders	5	9

## Discussion

Of the 189 drug products analyzed in our study, we focused on those package inserts that had the information categorized as “adverse reaction rate in PMO studies for re-examination,” and compared these to information described as “adverse reaction rate in clinical studies for NDA.” We found that, in 88.3 % of the 206 sets of information obtained for 176 drug products (182 of 206 sets), the adverse reaction rate in clinical studies for NDA was higher than that in PMO studies for re-examination (Fig. 1). On the contrary, as shown in Fig. 2, the adverse reaction rate in PMI studies for re-examination, which were conducted postmarketing, exhibited a profile similar to that found in clinical studies for NDA. Furthermore, as shown in Fig. 3, the proportion by which the incidence rate of the most common adverse reaction in clinical studies for NDA decreased postmarketing correlated to the proportion by which the incidence rate of the overall adverse reactions decreased postmarketing.

Results of this study indicated that, even in observational studies controlled by the pharmaceutical regulations in Japan—that is, PMOs—the adverse reaction rate was lower than that in intervention studies in most cases. In contrast, intervention studies conducted either prior to or after approval exhibited similar profiles in terms of adverse reaction rate. In addition, our findings suggest that one reason for a lower adverse reaction rate in PMO studies was that the number of reports of adverse reactions that had occurred frequently prior to approval decreased postmarketing; in other words, expected and common adverse reactions and non-serious adverse reactions were likely to be subject to underreporting.

In Japan, PMI studies for re-examination and clinical studies for NDA are conducted under Good Clinical Practice (GCP) Ministerial Ordinance (MHLW Ministerial Ordinance No. 28, issued March 27, 1997). However, GCP does not apply to PMO studies for re-examination even though these provide most of the data in the re-examination application. Therefore, unlike PMO studies for re-examination, PMI studies for re-examination are expected to have a level of quality that is equivalent to that of clinical studies for NDA and appear to have a similar safety profile. But even for PMO studies for re-examination, it is usually stipulated in study protocols that all adverse events (regardless of causality) that occur during a specified period following the administration of the drug product in question be reported, and there consequently should be no differences in the safety information collected between before and after approval. Nevertheless, one of the reasons that observational studies have a different safety profile than intervention studies conducted before or after approval appears to be the lack of a GCP requirement dictating activities, such as monitoring, that ensure reliability. Furthermore, PMO studies for re-examination are often so-called “3000-case studies.” In a questionnaire survey of physicians about PMO studies, 32 % of the respondents indicated that they believed “there is no scientific validity” and 43 % that “scientific validity is not required” of such studies (Japanese Association of Pharmaceutical Medicine 2010). These results suggest that low motivation of investigators at participating medical institutions also contributes to the underreporting.

Hazell and Saad et al. (2006) showed that the median underreporting rate across 37 studies (not including Japanese) was 94 % and the reason for not reporting included a lack of time, difficulty in accessing reporting form, etc. In Japan, each PMS study is conducted in accordance with GPSP. Nevertheless, the results of our investigation suggest that it is difficult to prevent underreporting even in the studies that, unlike spontaneous reports, are conducted under contracts signed between pharmaceutical companies and medical institutions as specified by regulations.

The limitations of our investigation include the small sample size of PMI studies for re-examination compared with that of PMO studies. Another limitation is that, in our investigation of underreporting, changes in all individual adverse reactions were not investigated; we only investigated the changes before versus after approval in the number of adverse reactions most frequently reported prior to approval.

In Japan, to date, the most common studies to collect safety information after drug approval have been PMO studies. Now, similar to the situation in the EU and United States, a guideline on RMP has existed in Japan since 2012. This guideline requires that companies applying for market approval submit a RMP that contains postmarketing pharmacovigilance and risk minimization plans, accounting for the potential risks of the drug product. In addition, a system allowing direct adverse reaction reporting by patients was introduced in March 2012 in Japan; patient adverse reaction reporting systems were introduced in 1993 in the United States and subsequently in Europe, emphasizing the importance of information reported directly by patients (Avery et al. 2011). Furthermore, similar to the Sentinel Initiative in the United States, a large-scale (tens of millions of persons) health care information database sentinel project was initiated in Japan in 2011. Thus, systems for safety information surveillance following approval have reached a major turning point.

There is a need for increased types of PMS activities, including those using large-scale health care information databases. One option might be to exclude expected and non-serious adverse reactions that have already been identified by clinical studies for NDA, and which are more likely to be underreported, from specific pharmacovigilance activities. For important potential adverse reactions, PMI studies should be proactively planned and conducted with a control group to identify the degree of risks. Conducting PMS studies only in specific medical institutions with quality systems in place would be another potential solution. Through such efforts, postmarketing safety data might be collected in a better and more efficient way to enhance patient safety.

## Conclusions

From the present study, it was confirmed that underreporting, generally considered as associated with observational studies, occurred in PMO studies for re-examination. Also, it was suggested that expected and common adverse reactions were likely to be subject to underreporting. Now systems for safety information surveillance following approval have reached a major turning point in Japan, and we need to seek a better and more efficient way to collect postmarketing safety data with increased types of PMS activities.
